# Targeted Diphtheria Toxin-Based Therapy: A Review Article

**DOI:** 10.3389/fmicb.2019.02340

**Published:** 2019-10-18

**Authors:** Fatemeh Shafiee, Marc G. Aucoin, Ali Jahanian-Najafabadi

**Affiliations:** ^1^Department of Pharmaceutical Biotechnology, School of Pharmacy and Pharmaceutical Sciences, Isfahan University of Medical Sciences, Isfahan, Iran; ^2^Department of Chemical Engineering, Faculty of Engineering, University of Waterloo, Waterloo, ON, Canada

**Keywords:** diphtheria toxin, immunotoxin, cancer, transcriptional targeting, fusion protein, bacterial toxin

## Abstract

Cancer remains one of the leading causes of death worldwide. Conventional therapeutic strategies usually offer limited specificity, resulting in severe side effects and toxicity to normal tissues. Targeted cancer therapy, on the other hand, can improve the therapeutic potential of anti-cancer agents and decrease unwanted side effects. Targeted applications of cytolethal bacterial toxins have been found to be especially useful for the specific eradication of cancer cells. Targeting is either mediated by peptides or by protein-targeting moieties, such as antibodies, antibody fragments, cell-penetrating peptides (CPPs), growth factors, or cytokines. Together with a toxin domain, these molecules are more commonly referred to as immunotoxins. Targeting can also be achieved through gene delivery and cell-specific expression of a toxin. Of the available cytolethal toxins, diphtheria toxin (DT) is one of the most frequently used for these strategies. Of the many DT-based therapeutic strategies investigated to date, two immunotoxins, Ontak^TM^ and Tagraxofusp^TM^, have gained FDA approval for clinical application. Despite some success with immunotoxins, suicide-gene therapy strategies, whereby controlled tumor-specific expression of DT is used for the eradication of malignant cells, are gaining prominence. The first part of this review focuses on DT-based immunotoxins, and it then discusses recent developments in tumor-specific expression of DT.

## Introduction

Too many deaths around the world can be attributed to one form of cancer or another. According to GLOBCAN, it is expected that the yearly death toll due to cancer will reach 16 million by 2040 (Ferlay et al., 2018). Along with deaths, it is associated with an incredible amount of pain and suffering, largely due to the non-specific nature of most conventional treatments. Conventional chemo- and radio-therapy strategies focus on mass cell killing, not limited to cancer cells (Padma, 2015). This non-specific toxicity to normal proliferating cells has resulted in low therapeutic indices and narrow therapeutic windows for conventional anti-cancer drugs (Ducry and Stump, 2010) and has encouraged the development of target-specific agents that are more effective and specific to tumor cells, lowering the probability of their affecting healthy tissues (He and Shi, 2014). Rapid progress in cancer research, including the determination of cancer-specific cellular processes and surface antigens, have allowed the combination of rational drug design and consideration of cancer biology for the development of novel targeted therapeutic strategies (Reiter, 2001; Kreitman, 2006).

The use of cell-surface molecules as recognition targets to selectively find and eliminate cancer cells while sparing normal cells is one approach for targeted cancer therapy (Denkberg et al., 2003). In this regard, a fusion protein consisting of a targeting moiety and an effector cytolethal moiety is produced. The targeting molecule is typically an antibody or antibody fragment or a ligand of a specific cell surface receptor that is either absent on the surface of normal cells or is highly up-regulated on cancer cells. Such a molecule is called an immunotoxin, especially when the targeting moiety is a molecule belonging to the immune system such as an antibody, cytokine, or growth factor (Jahanian-Najafabadi et al., 2012b).

Another strategy for the targeted killing of cancer cells is by delivering a gene of a cytolethal agent to cells and limiting its expression to within cancer cells through transcriptional regulation. This strategy, which might be called expression-targeting, uses specific transcriptional control elements such as enhancers or tumor-specific promoters to drive transcription of a particular gene in targeted cells (Fang et al., 2010; Javan and Shahbazi, 2017). Tumor-specific promoters are derived from genes with ectopic up-regulated levels of mRNA, and consequently protein, in cancer cells (Saukkonen and Hemminki, 2004). When these promoters are used upstream of a gene encoding for a toxic agent, no significant expression is expected to occur in normal cells even after taking up the expression cassette (Chen C. et al., 2018).

This review aims to highlight the advances in diphtheria toxin (DT)-based anti-cancer strategies as well as the targeting systems that are used to reduce non-specific destruction of non-cancerous cells. Specifically, the present manuscript will focus on DT or its derivatives as part of immunotoxins or targeted fusion proteins. We then review recent applications of the DT coding sequence used in DNA cassettes to mediate DT expression under the control of tumor-specific promoters. Tables 1, 2 summarize DT-based immunotoxins and gene therapy studies, respectively. Although we try to be as comprehensive as possible, we have limited our review mostly to those therapeutics that have advanced at least to *in vivo* studies.

**TABLE 1:** List of immunotoxins containing different truncated forms of DT attached to various targeting moieties for cancer therapy.

**Immunotoxin**	**Toxin/Toxin fragment**	**Targeting moiety**	**Target disease**	**Extent of studies**	**References**
DAB486IL-2	DT486	IL-2	NHL, HD, CTCL/Rheumatoid arthritis	Clinical trial	LeMaistre et al., 1993; Moreland et al., 1995
DAB389IL-2 (Denileukin Diftitox^TM^)	DT389	IL-2	NHL, HD, CTCL,	Clinical trial, FDA approved	Duvic et al., 2002
DT388-GM-CSF	DT388	GM-CSF	AML	Clinical trial	Frankel et al., 2002b
DT388-IL3	DT389	IL-3	AML, BPDCN	Clinical trial, FDA approved	Syed, 2019
DT389-EGF	DT389	EGF	Bladder cancer, lung cancer, glioblastoma	Clinical trial	Theodoulou et al., 1995
A-dmDT390-bisFV	Mutant DT390	A bispecific antibody against CD3	CTCL, PTCL, GVHD	Clinical trial	Frankel et al., 2015
DT2219	DT390	Bispecific scFv toward CD19 and CD22	B cell leukemia or lymphoma	Clinical trial	Bachanova et al., 2015
Tf-CRM107 (TransMID)	A mutated form of intact DT	Transferrin	Breast cancer, glioblastoma, medulloblastoma	Clinical trial	Yoon et al., 2010
DTAT/DTAT13/DTATEGF	DT390	AT fragment of uPA/AT-IL13/AT-EGF	Glioblastoma	Xenograft nude mice	Ramage et al., 2003; Hall and Vallera, 2006; Huang et al., 2012
DTEGF13	DT390	EGF and IL13	Glioblastoma, prostate, and pancreatic cancer	Xenograft nude mice	Oh et al., 2010
DT-antiCCR4	DT390	scFv against CCR4	T-cell malignancies	Xenograft nude mice, cynomolgus monkeys	Wang et al., 2016
DT386-BR2	DT386	BR2 (a cancer-specific CPP)	Breast cancer, leukemia, cervical cancer	*In vivo* safety assessment in albino mice	Shafiee et al., 2016
DT390-biTMTP1/DT390-triTMTP1	DT390	Double/Triple repeats of TMTP1synthetic pentapeptide (NVVRQ)	Highly metastatic cancer cells	Xenograft nude mice	Ma et al., 2013
DT-CD19	DT390	scFV against CD19	CD19^+^ lymphoma	Xenografted immunodeficient NSG mice	Zheng et al., 2017
DTIL13	DT389	IL-13	Gleioblastoma	Xenograft nude mice	Rustamzadeh et al., 2006
DT-SCF	DT387	stem cell factor	Ovarian, pancreatic, stomach, and liver cancers	*In vitro*	Potala and Verma, 2010
DT389-GRP	DT389	gastrin-releasing peptide	Brest cancer, prostate cancer, colon cancer	*In vitro*	Vanderspek et al., 1997
DAB389-IL7	DT389	IL-7	Hematopoietic malignancies	*In vitro*	Sweeney et al., 1998
DL_9_F and DL_2_F	DT389	Basic fibroblast growth factor	Ovarian teratocarcinoma	*In vitro*	Beitz et al., 1992; Zhang et al., 2006
DT389GCSF	DT389	Granulocyte-colony stimulating factor	G-CSF receptor-overexpressing cancer cells	Not evaluated	Babavalian et al., 2019

**TABLE 2:** Gene cassettes used to drive DTA expression within cancer cells.

**Expression cassette**	**Promoter**	**Target malignancy**	**Extent of studies**	**References**
pDTA-PBH19	814 bp flanking the 5′-region of the H19 gene	Bladder, choriocarcinoma, colorectal, and ovarian cancers	Clinical Trial	Lavie et al., 2017
pP3-DTA and pP4-DTA/pP4-DTA-P3-DTA	Insulin-like growth factor 2, promoters 3 and 4	Human bladder and hepatocellular carcinoma	Xenograft murine model	Ayesh et al., 2003; Amit et al., 2011
pAF-DTA	Alpha-fetoprotein promoter	Hepatocellular carcinomas and teratomas	Xenograft murine model	Kunitomi et al., 2000
pRAD51-DTA, pRAD51C-DTA, pXRCC2-DTA	Rad51, Rad51C, and XRCC2 regulatory elements	Various malignancies including breast and cervical cancers	Xenograft murine model	Hine et al., 2012; Cao et al., 2014; Chen Y. et al., 2018
pPSAR-PCPSA-DTA	Prostate-specific antigen promoter	Prostate cancer	Xenograft murine model, TRAMP mice	Zheng et al., 2003
Surp1430-DTA	Survivin promoter	Ovarian, gastric, non-small- and small-cell lung, and breast cancer	Xenograft murine model	Lin et al., 2015
V3-DTA	Heat shock promoter and upstream control element	Pancreatic cancer	Xenograft murine model	Fogar et al., 2010
phTERT-DTA	Human telomerase reverse transcriptase	Hepatocellular carcinoma, bladder carcinoma, and osteosarcoma	*In vitro*	Abdul-Ghani et al., 2000
CPT4-DTA	A synthetic β-catenin-dependent promoter	Hepatocellular carcinoma	*In vitro*	Lipinski et al., 2004
pTHA-47 or pTHA-49	Human chorionic gonadotropin α and β promoters	Breast and ovarian cancer	*In vitro*	Lidor et al., 1997
pTHA71	Immunoglobulin (Ig) kappa light chain gene regulatory sequences	B-cell malignancies	*In vitro*	Maxwell et al., 1992

## Immunotoxins

Immunotoxins are fusion proteins comprised of a toxic moiety and a targeting moiety, in which a known toxin molecule is usually truncated to remove its native binding domain, which is replaced by a targeting molecule that allows concentration of the fusion protein at the plasma membrane of specific cell types (Jahanian-Najafabadi et al., 2012b). The toxic moieties of immunotoxins have been derived from bacterial, fungal, plant, and even animal toxins (Cao et al., 2012; Weidle et al., 2014), while growth factors, monoclonal antibodies, cytokines (Aruna, 2006), and cancer-specific cell-penetrating peptides (CPPs) (Kersemans and Cornelissen, 2010; Soleimani et al., 2016, 2019) have been used as targeting moieties. Of the bacterial toxins, *Vibrio cholerae* toxin (Sarnovsky et al., 2010), Shiga toxin (Al-Jaufy et al., 1994; Jahanian-Najafabadi et al., 2012a), Pseudomonas exotoxin A (Yu et al., 2017; Dhillon, 2018), and DT (Vallera et al., 1999; Liu et al., 2000; Vaclavkova et al., 2006) have been used as either an immunotoxin or some other form of targeted toxin. Among these, DT is the most widely used due to its easy expression, high activity, and minimal side effects in humans (Brinkmann et al., 1995). Furthermore, there is detailed information on the three-dimensional structure of DT and its various fragments, which helps in the selection of appropriate peptide linkers to conjugate the toxin fragment to a targeting moiety as well as to maintain the activity of both parts (Choe et al., 1992; Louie et al., 1997).

## Diphtheria Toxin

**FIGURE 1:**
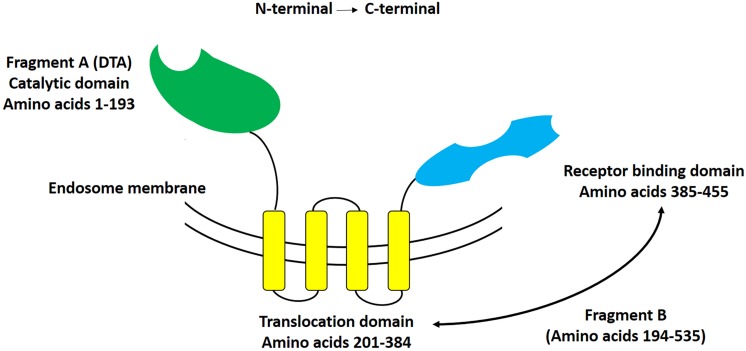
Schematic representation of diphtheria toxin. This Y-shaped molecule consists of two different fragments, that at the N-terminal side being named fragment A, and that at the C-terminal side being named fragment B. Fragment A includes the catalytic domain of DT, whereas fragment B includes both the translocation (T), and receptor-binding (R) domains of DT.

Diphtheria toxin is a single chain, 62 kDa protein consisting of 535 amino acid residues that is produced by *Corynebacterium diphtheria* containing lysogenic beta phage (Holmes, 2000). DT mediates its cytolethal effect through the inhibition of protein synthesis in susceptible cells (Bennett and Eisenberg, 1994). As it schematically represented in Figure 1, DT is a Y shaped molecule containing two functionally different regions: A and B. The A fragment (located at the N-terminus), includes a catalytic domain (C domain; 22 KDa, residues 1–193) that stops protein synthesis within eukaryotic cells. The B fragment (located at the C-terminus), on the other hand, consists of two domains, a transmembrane domain (T domain, 22 KDa, residues 201–384), and a receptor-binding domain (R domain, 18 KDa, residues 385–455).

**FIGURE 2:**
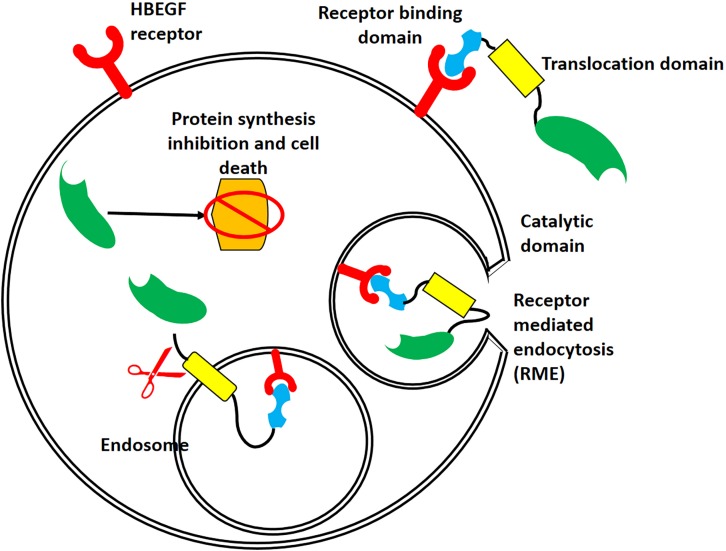
Interaction of DT with its receptor, followed by its internalization. After binding of DT to the heparin-binding epidermal growth factor receptor (HBEGFR, red), receptor-mediated endocytosis relocates DT to the cytosol. The acidic pH of the endosome causes conformational changes in the T domain (yellow) and membrane, resulting in a large channel that allows translocation of the C domain (green) and its release into the cytoplasm.

**FIGURE 3:**
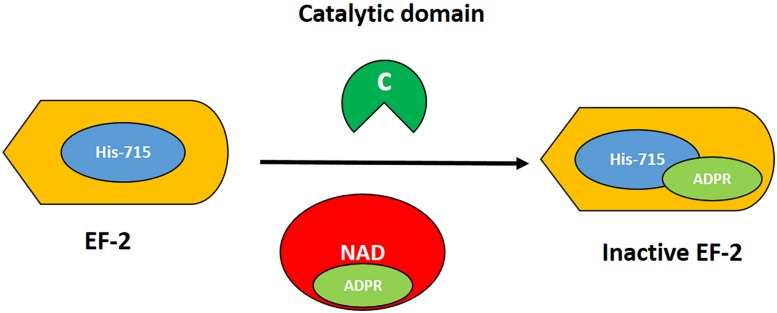
Mechanism of action of DT. The catalytic domain (green) acts by transferring the ADPR moiety (light green) from NAD (red) to the post-transcriptionally modified histidine residue at 715 (diphthamide; blue) of elongation factor 2 (EF2, orange). Thus, the EF2 is irreversibly inactivated, resulting in inhibition of protein synthesis and cell death.

The T domain helps in the translocation of the C domain from the endosome to the cytosol, while the R domain helps to bind the heparin-binding epidermal growth factor receptor (HBEGFR) on the surfaces of susceptible cells (Bennett and Eisenberg, 1994). In the cytoplasm of susceptible cells, the catalytic domain first binds to nicotinamide dinucleotide (NAD) and then transfers an adenosine diphosphate ribosyl (ADPR) moiety to elongation factor 2, which subsequently inhibits protein synthesis (Collier, 2001). The interaction of DT with its cell surface receptor and its mechanism of action are summarized in Figures 2, 3, respectively.

## Diphtheria Toxin-Based Immunotoxins

Truncated forms of DT have been successfully used to generate recombinant immunotoxins against cancer (Shapira and Benhar, 2010). Genetic substitution of the native DT R domain with cytokines, growth factors, cancer-specific CPPs, and generally any ligands specific for cancer cell antigens have resulted in the formation of fusion proteins that retained the functions and activities of their constituent parts (Beilhartz et al., 2017). Table 3 represents most of the cell surface receptors or antigens targeted for construction of DT-based immunotoxins to date. *In vitro*, these immunotoxins have shown very small IC_50_s on the order of 10^–9^–10^–14^ M, which validates their possible use as a targeted therapy. For cells that are not targeted or that do not contain a targeted receptor, DT-based immunotoxins tend to have an IC_50_ on the order of 10^–7^ M.

**TABLE 3:** Cell surface receptors used as targets for DT-based immunotoxins and their presence in malignancies and normal tissue.

**Targeted receptor or antigen**	**Targeted malignancies**	**Normal tissue**	**References**
IL2 receptor	Some T-cell malignancies	T cells	Zachariae et al., 1991
GM-CSF receptor	Lymphoma	Multipotent myeloid progenitor cells, monocytes, DCs, macrophages, and neutrophils	Rozemuller et al., 1997; Westcott et al., 2004; Rosas et al., 2007
EGF receptor	Different malignancies including breast, lung, bladder, head-and-neck, and pancreatic cancers	Low expression in various normal tissues	Simon and Fitzgerald, 2016
IL3 receptor	Lymphoma	Multipotent and committed myeloid and lymphoid progenitors	Suda et al., 1985; Park et al., 1989
CD3	Malignant T-cells	T-cells	Frank et al., 1991
CD19	B cell leukemia or lymphoma	Late pre-B cells and B cells	Uckun et al., 1988
CD22	B cell leukemia or lymphoma	Pro-B cells and mature B cells	Sato et al., 1996
Transferin receptors (TfRs)	Various tumors	Low-level expression at most human tissues	Hagihara et al., 2000
uPA receptor	Many cancers including fibrosarcoma, melanoma, breast, prostate and colon carcinoma, leukemia	Many cell types including monocytes, granulocytes, endothelial cells, fibroblasts, keratocytes, and hepatocytes	Lanza et al., 1998
IL13 receptor	Renal cell carcinoma, malignant glioma, ovarian carcinoma, and squamous cell carcinoma of head and neck	Normal immune cells and tissues express very low levels	Joshi and Puri, 2009
CCR4	T-cell leukemia-lymphoma, adult peripheral T-cell lymphoma, T-cell acute lymphoblastic leukemia, and cutaneous T cell lymphoma	Effector Treg cells, and rare expression by CD8+ T cells, NK cells, CD14+ monocytes/macrophages, dendritic cells, and B cells	Sugiyama et al., 2013

### DAB486IL-2 and DAB389IL-2

Interleukin-2 (IL-2) is a cytokine that is primarily produced by CD4+ T-cells following their activation by antigens. Three different IL-2 receptor chains exist that together generate low-, intermediate-, and high-affinity IL-2 receptors. The ligand-specific IL-2 receptor α chain (IL-2Rα, Tac antigen) binds IL-2 with low affinity, the combination of IL-2Rβ and IL-2Rγc together forms an IL-2Rβ/γc complex that binds IL-2 with intermediate affinity, and when all three receptor chains are co-expressed, IL-2 is bound with high affinity (Kim et al., 2006). Some T-cell malignancies overexpress the IL2R, making it an ideal target for immunotoxins (Urba et al., 1990; Zachariae et al., 1991).

DAB486IL-2, a 68 kDa-recombinant protein, showed selective binding affinity to cells expressing high-affinity IL-2R (Williams et al., 1987, 1990). C91/PL, C8215, and CTLL2, which are IL-2R-positive cells, have been shown to be highly sensitive to this recombinant protein, with IC_50_s ranging 0.1–50 × 10^–10^ M, whereas IL-2 negative cell lines had IC_50_s greater than 10^–7^ M (Bacha et al., 1988; Waters et al., 1990, 1991).

DAB486IL-2 advanced to a phase I clinical trial for treatment of chemotherapy-resistant hematological malignancies. From 18 evaluable patients in the study, the maximum tolerated dose (MTD) was determined to be 100 μg/kg/day given as an intravenous (IV) bolus over 10 days. The main adverse effect was reversible elevations in hepatic transaminases. Other mild reversible side-effects included rash, nausea, elevated creatinine, chest tightness, and fever (LeMaistre et al., 1992). Furthermore, the MTD when infused for 90-min daily for five consecutive days was slightly higher, at 300 μg/kg/day, and higher doses resulted in renal insufficiency associated with hemolysis and thrombocytopenia (LeMaistre et al., 1993).

DAB486IL-2 has also advanced to clinical trials for treatment of rheumatoid arthritis (RA). In an open-label, phase I/II clinical trial on patients with RA refractory to methotrexate, nine patients showed a substantial or meaningful response. The MTD for patients receiving DT486IL-2 for 5 to 7 consecutive days was 0.1 mg/kg/day (Sewell et al., 1993), similar to the studies for the treatment of chemotherapy-resistant hematological malignancies. The anti-arthritic effect of this fusion protein was further evaluated in a subsequent pilot phase II, double-blind, placebo-controlled trial on 45 RA patients, and 18% of patients showed significant clinical improvement (Moreland et al., 1995).

To improve the short half-life of DT486IL-2, which is on the order of 5 min (Bacha et al., 1990), and to reduce the immunogenicity of the molecule, shorter DT fragments were investigated by removing the remaining hydrophobic sequences of its binding domain (Chaudhary et al., 1991; Kuzel et al., 1993). DAB389IL-2, which is a 58 kDa variant containing 97 fewer amino acids than its parent molecule, DAB486IL-2, showed a fivefold improvement in affinity for the IL-2R and an approximately 10-fold increase in cytotoxicity toward IL-2R-positive cancer cells (Bacha et al., 1991; Kiyokawa et al., 1991). Cell lines of hematopoietic malignancies expressing the high-affinity IL-2R displayed IC_50_s of 10^–12^ to 10^–11^ M. Cells exhibiting an intermediate-affinity receptor had IC_50_s on the order of 10^–10^ to 10^–9^ M, and cells with low-affinity IL-2R had IC_50_ values greater than 10^–7^ M (Bacha et al., 1991; Re et al., 1996). DAB389IL-2 has also shown an improved half-life of 72 min (LeMaistre et al., 1998).

In addition to IL-2R-positive malignancies, the effectiveness of DAB389IL-2 was also shown *in vitro* for the suppression of immunopathology in schistosomiasis (Ramadan et al., 1995) and HIV infection (Zhang et al., 1997) and also in clinical trials against psoriasis. For the latter, a phase I clinical study showed moderate to large improvement in 8 out of 10 psoriatic patients that received systemic injections of DAB389IL-2 (Gottlieb et al., 1995). However, despite promising antipsoriatic activity, a further phase II clinical study revealed high toxicity at the required doses and schedules for routine treatment of psoriasis (Bagel et al., 1998).

It is, however, with the IL-2R-positive malignancies that DAB389IL-2 has had the most encouraging results. Phase I/II clinical trials on relapsed cutaneous T-cell lymphoma (CTCL), non-Hodgkin’s lymphoma (NHL), and Hodgkin’s disease (HD) patients with confirmed expression of either p55 or p75 IL-2R subunits verified the activity and safety of DAB389IL-2. The MTD for patients that received up to eight courses of short IV infusions for 5 consecutive days, repeated every 2 days, was 27 μg/kg/day. At 31 μg/kg/day, patients became asthenic, which was considered the dose-limiting toxicity. Of the 73 patients included in the study (35 with CTCL, 17 with NHL, and 21 with HD), five complete and eight partial remissions (CR and PR, respectively) were obtained in CTCL patients; and one CR and two PRs were observed in NHL patients. No responses were observed in patients with HD (LeMaistre et al., 1998; Saleh et al., 1998). Further clinical studies of DAB389-IL2, including a phase III clinical study on 71 patients with biopsy-proven CTCL expressing the IL-2R (Olsen et al., 2001; Duvic et al., 2002) led to its approval by the FDA for the treatment of persistent or recurrent CTCL under the name denileukin diftitox (ONTAK^TM^) in 2008.

Unfortunately, ONTAK^TM^ was discontinued in 2014 due to issues related to the production of the immunotoxin in Escherichia *coli* (Wang et al., 2017). DAB389IL-2 was revived as a better-formulated drug now referred to as E7777. E7777, which has improved purity and an increased percentage of active protein, is 1.5–2 times more bioactive than ONTAK^TM^ (Duvic et al., 2014), and a phase I clinical trial on Japanese patients with relapsed or refractory peripheral and cutaneous T-cell lymphoma revealed an MTD of 9 μg/kg/day (Maruyama et al., 2015; Ohmachi et al., 2018). Phase II and phase III clinical trials of E7777 are currently ongoing (ClinicalTrials.gov identifiers: NCT02676778 and NCT01871727, respectively).

Other attempts to improve the production or enhance the potency of DAB389IL-2 have also been reported. In a recent study, production and evaluation of a bivalent ONTAK^TM^-like (DAB389-IL2IL2) protein in a DT resistant *Pichia pastoris* with 100-fold higher *in vitro* potency and comparable *in vivo* efficiency has been reported. However, this immunotoxin has not been evaluated clinically yet (Peraino et al., 2014; Wang et al., 2017). Another group also reported the expression and purification of a secreted biologically active Val6Ala mutant form of the DAB389IL2 [s-DAB-IL2(V6A)] in *C. diphtheria*. The mutation reduced vascular leaks, a usual side effect of DAB389IL2, and increased the MTD. The LD50 of the mutated molecule was 3.7-fold higher than DAB389IL-2 in mice. Expression in *C. diphtheria* also reduced the aggregation problems observed for ONTAK^TM^ (Cheung et al., 2019). There is, therefore, still significant potential for an IL-2-targeting DT immunotoxin as a therapeutic approach.

### DT388GMCSF

Granulocyte-macrophage colony-stimulating factor (GM-CSF) is a monomeric glycoprotein cytokine involved in the maturation and proliferation of white blood cells. Its receptor, GMCSFR, is overexpressed in leukemic cells, which could be targeted with GM-CSF (Rozemuller et al., 1997).

DT388GMCSF contains the C and T domains of DT fused to human GM-CSF. The IC_50_ of this immunotoxin against cell lines overexpressing GMCSFR such as HL60, U937, and TF1 was on the order of 10^–12^ M (Frankel et al., 1997). It has also been cytotoxic to chemotherapy-resistant cell lines, and on therapy-refractory progenitor cells separated from acute myeloid leukemia (AML) patients without being toxic to normal human myeloid progenitor cells (Hogge et al., 1998). In a preclinical *in vivo* study involving a xenograft model of human AML, anti-leukemic effects were shown through the treatment of abdominal masses formed from HL60 cells. The treatment consisted of 84 μg/kg/day of DT388GMCSF intraperitoneally injected on days 2 through 6, which showed to be the MTD in mice (Hall et al., 1999). In cynomolgus monkeys, however, an 11-fold lower MTD (7.5 μg/kg/day) was observed compared to mice. It was suggested that cross-reactivity of receptors in monkeys led to distinct dose-limiting toxicities (Hotchkiss et al., 1999).

Finally, in a phase I clinical trial involving AML patients, 15 min IV infusions for 5 consecutive days showed significant remission in patients. The MTD was determined to be 4 μg/kg/day. At doses around 4.5–5 μg/kg/day, liver injury was reported. Among nine patients treated at those doses, one patient developed liver failure, and one patient had transient hepatic encephalopathy. Although DT388GMCSF did not show any *in vivo* damaging effect on hepatic cell lines, the correlation of increased DT388GMCSF serum concentrations and increased serum aspartate aminotransferase levels, along with DT388GMCSF-induced cytokine release by macrophages, halted further clinical applications (Frankel et al., 2002b). A follow-up to determine the mechanism of the observed hepatotoxicity with a murine version of the fusion protein, DT390mGMCSF, showed that the observed hepatotoxicity was not a result of non-specific uptake but rather of the uptake of the fusion protein by GMCSF-receptor-positive hepatic macrophages (Kupffer cells), which causes their depletion (Westcott et al., 2004). This observation limited further studies on the production and application of GMCSFR-based immunotoxins.

### DT388-IL3

Interleukin 3 (IL-3) is a growth factor that induces the proliferation and terminal differentiation of multipotent and committed myeloid and lymphoid progenitors without affecting hematopoietic stem cells (Suda et al., 1985). The IL-3 receptor (IL3R) consists of an α and a β subunit. While the α subunit is unique to the IL3R and is the site of ligand attachment and represents the specificity of the receptor, the β is a subunit shared with the GM-CSF and functions in signal transduction and internalization of the ligand–receptor complexes (Park et al., 1989).

Similar to DT388GMCSF, DT388-IL3 was developed to treat AML. A preliminary *in vitro* and *in vivo* (C57BL/6 mice) study using DT390mIL-3 (DT390 fused to mouse interleukin 3) confirmed the potential for application of IL-3 to target AML (Chan et al., 1996). DT388-IL3 was subsequently developed and showed cytolethal activity on different IL-3R-overexpressing human leukemia cell lines, with IC_50_ values ranging from 1 to 28 × 10^–12^ M. Receptor-negative cell lines were not impaired at concentrations exceeding 1.4 × 10^–9^ M (Frankel et al., 2000). Unlike DT388GMCSF, which showed a modest but significant toxicity against normal progenitor cell types (Feuring-Buske et al., 2000), a study on non-obese diabetic/severe combined immunodeficient (NOD/SCID) mice inoculated with human IL-3-receptor-positive AML blasts receiving DT388IL3 at daily doses of 0.045 μg/g for 5 consecutive days revealed no significant toxicity against equivalent normal cells, while it resulted in an up to 83% size reduction in AML engraftments. In addition, no evidence of leukemia was detected in two of the samples after 12 weeks (Feuring-Buske et al., 2002). Further toxicology and pharmacokinetic studies in cynomolgus monkeys revealed an MTD of 100 μg/kg/day when injected every other day over 12 days. At 150 μg/kg/day, subjects showed elevated liver enzyme concentrations, severe malaise and anorexia (Cohen et al., 2004, 2005).

A phase I clinical trial on 45 patients (40 AML and 5 myelodysplasia patients) established an MTD but had only moderate success. Patients were scheduled to receive one of five dose levels between 4 and 12.5 μg/kg/day as 15 min IV infusions every other day over 12 days. Due to the prolonged infusion schedule, many patients failed to receive the 6 doses. Still, the MTD was determined to be > 12.5 μg/kg/day. Of the AML patients, one showed CR for 8 months and one showed PR for 3 months. Of the myelodysplasia patients, one showed PR for 4 months (Frankel et al., 2008).

A subsequent phase I/II clinical trial on 11 patients with blastic plasmacytoid dendritic cell neoplasm (BPDCN), however, established a role for DT388-IL3 in the management of BPDCN and other hematologic malignancies. BPDCN patients received 12.5 μg/kg/day DT388-IL3 (aka SL-401) IV over 15 min for up to 5 days. Three patients with an initial response to SL-401 received a second course in relapse. Seven of nine evaluable BPDCN patients had major responses, including five CR and two PR after a single course of SL-401 (Frankel et al., 2014). Further clinical and non-clinical evaluations of this immunotoxin (Pemmaraju et al., 2017; Sun et al., 2018) led to its approval by the FDA in 2018 for the treatment of adult and pediatric BPDCN under the name of Tagraxofusp^TM^ (Syed, 2019).

### DAB389EGF and DAB486EGF

Another growth factor whose receptor is overexpressed in cancer cells is the EGF. It has been reported that the EGF receptor (EGFR) has a more than 320-fold overexpression in some human cancer cell lines (Pfeiffer et al., 1990). Preliminary *in vitro* studies on A431 (vulval carcinoma), KB (oral carcinoma), and A549 (lung adenocarcinoma) cell lines revealed a 10- to 100-fold higher potency for DAB389EGF over DAB486EGF, consistent with the findings for the DT-IL2 immunotoxins. IC_50_ values for DAB389EGF and DAB486EGF on A431 cells were 2 × 10^–12^ M and 1 × 10^–10^ M, respectively (Shaw et al., 1991). In addition, IC_50_ values ranging from 4 × 10^–13^ to 5 × 10^–12^ M were determined on 13 different human glioma cell lines overexpressing EGFR (Liu et al., 2003).

Preclinical animal studies on DAB389EGF in rats revealed an MTD of 30 μg/kg/day upon receiving daily bolus IV infusions for 10–14 days. Renal and hepatic injury was observed at higher doses (Cohen et al., 2003). Further preclinical animal studies on cynomolgus monkeys revealed a lower MTD of 20 μg/kg/day for 10 days, which is similar to that for the DT388GMSCF immunotoxin, potentially due to the cross-reactivity of receptors in monkeys. Again, hepatic and renal injuries were the dose-limiting toxicities in the studied monkeys. Afterward, the *in vivo* efficacy of the immunotoxin was evaluated in nude mice inoculated with human EGFR-positive A549 lung adenocarcinoma cells. Treatment of the xenografts with 25 μg/kg/day for 10 days resulted in 50% tumor growth inhibition and also complete tumor regression in 20% of the mice (Theodoulou et al., 1995). By considering the promising effect of DAB389EGF in the xenograft animal model, phase I/II clinical trials were carried out with bolus IV infusions of DAB389EGF over 1–3 weeks in 72 patients with EGFR-positive metastatic carcinomas. However, the results of the clinical trials showed only a 6-month partial remission in one patient with non-small cell lung carcinoma. The MTD was 6 μg/kg/day, with renal and liver injuries and chest pain as the most significant dose-limiting toxicities. Therefore, clinical study on this immunotoxin was discontinued (Theodoulou et al., 1995).

Recently, targeting the EGFR receptor with the conventional DAB389EGF immunotoxin or different DT fusion constructs has gained new research attention. In one study, Yang et al. (2013) evaluated the cytotoxicity of DAB389EGF on various bladder carcinoma cell lines. Their results revealed that the immunotoxin had a potent cytolethal effect on all cell lines including J82, RT4, CRL1749 (CRL), T24, TCCSUP (SUP), and HTB9, with IC_50_ values ranging from 3 × 10^–11^ to 1 × 10^–10^ M, while the IC_50_ was higher than 1 × 10^–9^ M for the EGFR-negative H520 control cell line. Next, they induced xenograft bladder tumors in C57BL/6 female nude mice by instilling 100 μL of 1.5 × 10^6^ HTB9-Luc cells (HTB9 cells stably transfected with a luciferase-expressing cassette for simpler *in vivo* imaging of tumor size changes) into the animal bladder. Once tumor implantation was confirmed at day 7, 70 μl of 1 μg/μl of the immunotoxin solution was injected into the animals’ bladder. This intravesical treatment was repeated twice per week for 2 weeks. This study revealed a subjective decrease in luminescent activity after 1 week and a nearly uniform loss of luminescent activity after 2 weeks of treatment. In addition, no obvious behavioral or organ and tissue toxicity was observed in the treated animals, showing that, in contrast to the systemic administration of the DT386EGF, its intratumoral administration could be safe.

A variation on targeting EGFR is targeting EGFRvIII (de2-7 EGFR), a tumor-specific receptor produced by 801 bp in-frame deletion of the EGFR nucleotide sequence through alternative splicing. This receptor lacks 267 amino acids in the extracellular domain and also has a new glycine residue and unique peptide sequence at the splice site (Humphrey et al., 1990). EGFRvIII could not bind EGF but has constitutive kinase activity, resulting in hyperactivation of growth, promoting signaling and enhanced proliferation of the EGFRvIII-bearing cells (Nagane et al., 1996). EGFRvIII has been targeted using a novel immunotoxin, DT390-BiscFv806, composed of DT390 and a bivalent single-chain Fv antibody fragment derived from a mouse monoclonal anti-EGFRvIII antibody, Mab 806. *In vitro*, EGFRvIII-transfected U87 (U87-EGFRvIII) glioblastoma cells showed to be significantly more sensitive to DT390-BiscFv806, with an IC_50_ of 2.26 × 10^–13^ M, compared to U87 cells (IC_50_ 1.47 × 10^–9^ M). *In vivo*, DT390-BiscFv806 significantly reduced tumor size in both U87 and U87-EGFRvIII xenograft mouse models (Meng et al., 2015). To date, there no clinical information has been reported for DT390-BiscFv806.

### A-dmDT390-bisFV

CD3 is a multimeric protein complex that functions as a T-cell co-receptor. Due to the lack of signaling capability in the cytoplasmic portion of the T-cell receptor (TCR), T-cell activation via TCR depends on the CD3 protein complex. CD3 is expressed on normal and malignant T-cells at their earliest developmental stages, so its targeting could be useful for the treatment of T-lineage neoplasms (Frank et al., 1991). CD3 was first targeted within the context of immunotoxins as an immunosuppressive agent to combat graft-versus-host disease. Primary attempts used either intact ricin A (RA) or DT bound to an intact anti-human CD3 murine monoclonal antibody (UCHT1). *In vitro* studies on human peripheral T-cells and T-leukemia cells (Jurkat cells) revealed that a 10–100 times lower concentration of UCHT1-DT was needed compared to UCHT1-RA to have an effect. UCHT1-DT also showed faster onset and greater selectivity between T-cells and stem cells than did UCHT1-RA (Youle et al., 1986). However, further *in vivo* mouse studies showed that intact anti-CD3 monoclonal antibodies activated T-cells via their Fc fragment and resulted in more non-specific toxicity than the immunotoxin made with an antibody fragment (Blazar et al., 1991; Vallera et al., 1995). In addition, another study showed that an immunotoxin made from a single-chain antibody and DT390 (DT390scFv) retained its cytotoxicity on T-cells, with an IC_50_ of about 4.8 × 10^–11^ M. Although this smaller immunotoxin showed 16 times less cytotoxicity than the intact UCHT1-derived immunotoxin (which had an IC_50_ of 2.9 × 10^–12^ M on T-cells), it was less affected by pre-existing neutralizing antibodies *in vivo* (Thompson et al., 1995). Meanwhile, an anti-monkey CD3 antibody (FN18) fused to intact (FN18-CRM9) or truncated (DT390-FN18sFv) DT yielded similar results, with the intact DT having better IC_50_ values (1 × 10^–10^ M) compared to the truncated version (1 × 10^–8^ M) (Knechtle et al., 1997; Ma et al., 1997).

A challenge with antibody- or antibody fragment-based immunotoxins is their production. In bacterial platforms such as recombinant *E. coli*, aggregation and subsequent solubilization and purification have been issues. These issues, however, are minimized by producing the protein in a higher eukaryotic platform. CHO cells have been a *de facto* platform for therapeutic antibody production; however, CHO cells are sensitive to the DT. CHO K1 RE1.22c cells have been used to overcome this challenge and obtain correctly folded DT390-scFv (Liu et al., 2000). These cells were mutated to become ADP-ribosylation insensitive. Although the cells were able to produce and secrete the immunotoxin, the cytotoxicity of the immunotoxin toward target cells was reduced. Further investigation revealed N-glycosylation of the DT390 fragment at two sites (at N-glycosylation motifs 16-18 and 235-237). Mutations (Ser18Ala, and Asn235Ala) to remove these N-glycosylation sites increased the IC_50_ values from 4.8 × 10^–10^ M (N-glycosylated) to 4 × 10^–12^ M (Non-glycosylated, A-dmDT390-scFv) on Jurkat cells. A-dmDT390-scFv was 12 times more effective than the DT390-scFv immunotoxin expressed by *E. coli* cells (Liu et al., 2000). Subsequent *in vitro* studies showed 10-fold higher cytotoxicity by a bivalent single-chain fragment immunotoxin, A-dmDT390-bisFv. *In vivo* studies in transgenic heterozygote mice (tgE 600) expressing either human or mouse CD3 antigen on the surfaces of their T-cells showed that the bivalent immunotoxin had a 9- and 34-fold higher potency for depleting spleen and lymph node T-cells, respectively, compared to the monovalent immunotoxin (Thompson et al., 2001). In addition, a larger derivative of the immunotoxin is expected to have lower kidney clearance and accumulation, as reported before (Vallera et al., 2000, 2005b). This results in decreased renal toxicity and failure, a dose-limiting toxicity, which had been shown in a preclinical murine study on monovalent DT390anti-CD3sFv (Vallera et al., 1997).

Later, since the production yield of the A-dmDT390-bisFv in the mutated CHO cell line was very low (2–5 μg/ml), which was not sufficient to go through different phases of the clinical trials, *Pichia pastoris* was used as a production host, which finally gave a production yield of 37 μg/ml in an optimized expression condition (Woo et al., 2002, 2004). Further preclinical studies on the A-dmDT390-bisFv produced in *Pichia pastoris* revealed an IC_50_ of 1.7 × 10^–14^ M on CD3-positive Jurkat Cells. In addition, the LD_10_ (the dose resulting in 10% death) for 10-week-old Balb/C female mice was between 500 and 750 μg/kg (cumulative after eight injections administered twice daily for 4 days). Renal tubular necrosis was the dose-limiting toxicity (Woo et al., 2008b). Another animal study in rats and squirrel monkeys was performed to refine the pharmacokinetics and pharmacodynamics. In this study, an MTD of 200 μg/kg was reported. This amount was sufficient for antitumor activity *in vitro* as well as in the rat model. However, in order to maintain CD3 occupation of lower than 10% (to prevent any T-cell activation and cytokine release), doses of 2.5 μg/kg twice daily for 4 days (20 μg/kg total) was used as the starting dose of the first-in-human clinical trial of A-dmDT390-bisFv (Resimmune^TM^) (Woo et al., 2008a). At those doses, in another primary phase I clinical trial, this one for CTCL patients, two PRs for 1 and 6 months were achieved. However, mild to moderate toxicities including fever, chills, nausea, transaminasemia, hypoalbuminemia, lymphopenia, and reactivation of the Epstein- Barr virus (EBV) and cytomegalovirus (CMV) were reported (Frankel et al., 2009). Recent updates on a Phase I/II clinical trial (http://clinicaltrials.gov identifier: NCT00611208) on 30 patients (including 25 with CTCL and three with peripheral T-cell lymphoma (PTCL)) showed responses for 36% of the CTCL patients, including four CR (ranging from 38 to 72 months) and five PR (ranging from 3 to 14 months). Results from Resimmune^TM^ are promising, and further studies in patients with low tumor burden stage IB-IIB CTCL is warranted (Frankel et al., 2015).

### DT2219

CD19 is a surface glycoprotein that is mainly expressed on late pre-B cells and B cells. It is also widely expressed on B cell leukemia or lymphoma including B-lineage lymphoblastic leukemia, which is the most common form of childhood leukemia (Anderson et al., 1984; Uckun et al., 1988). CD22 is a key regulatory cytoplasmic protein that is primarily expressed on pro-B cells and is then expressed simultaneously with immunoglobulin D as a surface membrane receptor on most mature B cells. CD22 is also expressed on the surface of ∼70% of B-cell lymphomas and leukemias (Peaker and Neuberger, 1993; Sato et al., 1996).

DT2219 is a bispecific immunotoxin consisting of DT390 fused to single-chain antibody fragments targeted to the CD22 and CD19 antigens. DT2219 came about following studies on HD37-dgRTA and RFB4-dgRTA, which were conjugates of deglycosylated ricin A toxin and full murine monoclonal antibodies against CD19 or CD22, respectively. These immunotoxins proved to be effective in patients with B cell lymphoma in separate clinical trials (Conry et al., 1995; Stone et al., 1996). Combotox, an equimolar combination of the two immunotoxins, led to an even higher survival rate for xenograft SCID mice inoculated with the pre-B acute lymphoblastic leukemia cell line, NALM-6-UM1 (Herrera et al., 2000, 2003). Considering the disadvantages of simultaneous administration of two immunotoxins, especially with regards to the increased rate of side effects, and also to broaden the reactivity against B-cell leukemias and lymphomas, Vallera et al. (2005a) developed DT2219. On the CD19^+^CD22^+^ Daudi cell line, this immunotoxin had the lowest IC_50_ (2 × 10^–9^ M) of different combinations tested (DT-sFvCD19, DT-sFvCD19sFvCD19, DT-sFvCD22, and DT-sFvCD22sFvCD22). This was more than one order of magnitude smaller than the next most potent immunotoxin tested, DT-sFvCD19sFvCD19, which had an IC_50_ of 3.2 × 10^–8^ M (Vallera et al., 2005a). Mutations in three amino acids through site-directed mutagenesis of the anti-CD22 variable heavy region enhanced avidity toward the CD22 antigen and lowered the IC_50_ to 3 × 10^–10^ M. *In vivo* evaluation in xenograft mice with established flank Daudi tumors resulted in significant survival of mice that received four 20 μg doses on days 12, 13, 14, and 16 post tumor inoculation. In SCID mice made to have systemic leukemia tumors through the injection of Daudi cells, those that were treated with 20 μg of DT2219 on days 3, 6, 15, 19, 26, 31, 42, 45, and 49 post inoculation resulted in significant animal survival compared to the controls (Salvatore et al., 2002; Vallera et al., 2005a). DT2219 was further improved by reversing the orientation of the VH and VL chains and fusing each sFv to DT390 via an aggregation reducing/stabilizing linker (ARL: GSTSGSGKPGSGEGSTKG). These changes improved the IC_50_ values to between 6 × 10^–11^ and 2 × 10^–10^ M. *In vivo*, xenograft mice inoculated with human Raji Burkitt’s lymphoma cells treated with the improved DT2219 (now known as DT2219ARL) showed long-term tumor-free survivors. The MTD in rabbits receiving IV injections was 200 μg/kg of DT2219ARL when administered every other day over 7 days. At 500 μg/kg under the same regimen, hepatic failure was reported as the dose-limiting toxicity (Vallera et al., 2009). A phase I dose-escalation study to determine the safety, MTD, and efficacy of DT2219ARL included 25 patients with mature or precursor B-cell lymphoid malignancies expressing CD19 and/or CD22 antigens. The patients received DT2219ARL doses ranging from 0.5 μg/kg/day (about 1/500th of the MTD in rabbits) to 80 μg/kg/day intravenously (IV) every other day for 4 total doses (days 1, 3, 5, and 8). Clinical responses were observed at doses of 40 and 60 μg/kg/day, though the 4 doses were inadequate to induce deeper remission. However, one patient who achieved PR after 1 cycle was subjected to an additional cycle, and this led to complete tumor elimination. The safety and biological activity of DT2219ARL at doses of 40–80 μg/kg/day were subsequently approved (Bachanova et al., 2015), and the evaluation of the immunotoxin has proceeded to a phase I/II clinical trial (http://Clinicaltrials.gov identifier: NCT02370160). Although the completion of the study was slated for April 2018, there are still no reported data regarding the outcome of this study.

### Tf-CRM107 (TransMID)

Transferrin is an iron-binding glycoprotein that controls the level of free iron found in blood plasma. Receptors for transferrin (TfRs) are overexpressed on various tumors, including carcinomas, glioblastomas, and medulloblastomas (Daniels et al., 2012).

Transferrin-CRM107 (Tf-CRM107) is unique among the immunotoxins discussed thus far. It is constructed by chemically coupling human transferrin to a mutant whole DT structure (S525F). The mutation reduced the native binding activity of the toxin 8,000-fold, but maintained intact translocation and catalytic functions. Conjugation of transferrin and the mutated DT was achieved by thioesterification of the two proteins (Greenfield et al., 1987). *In vitro* studies determined the IC_50_ values for Tf-CRM107 against T47D (breast carcinoma), SNB40 (human medulloblastoma), and U251 (Human glioblastoma astrocytoma) cells to be between 0.39 and 2.6 × 10^–12^ M. In nude mice with human gliomas (U251), significant tumor reduction was achieved when treated with Tf-CRM107 (Johnson et al., 1988; Laske et al., 1994; Hagihara et al., 2000). The findings of phase I/II clinical trial demonstrated its tumor-suppressive capacity in 35% of patients with malignant brain tumors refractory to conventional therapies, without severe neurologic or systemic toxicity (Weaver and Laske, 2003). However, despite promising results from phase I and II clinical trials that showed positive effects in glioblastoma patients, the results of a phase III clinical trial determined that Tf-CRM107 was unlikely to improve overall patient survival compared to the current standard of care (Yoon et al., 2010).

### DTAT, DTAT13, and DTATEGF

Urokinase-type plasminogen activator (uPA) is a serine protease that catalyzes the conversion of plasminogen to plasmin. uPA interacts with the urokinase receptor (uPAR, aka CD87) to restrict plasminogen activation. In certain cancer cells such as glioblastoma cells uPAR is overexpressed (Mori et al., 2000). The IL-13 receptor has also been reported to be overexpressed on glioblastoma cells and minimally expressed on normal healthy cells. It has also been reported to be overexpressed in prostate and pancreatic cancer cells (Debinski et al., 1999; Li C. et al., 2002).

DTAT is an immunotoxin composed of the amino-terminal (AT) fragment of the uPA fused to the C-terminus of DT390. On U118MG, U373MG, and U87MG glioblastoma cells, DTAT had IC_50_s < 1 × 10^–9^ M. Treatment of nude mice bearing U118MG human tumors with five 20 μg/day doses administered every other day resulted in a significant regression of tumor sizes. Similar doses in healthy C57BL/6 mice did not affect any tissues or organs (Vallera et al., 2002). On uPAR-positive AML blasts obtained from patients, the IC_50_ for DTAT was lower than 1 × 10^–9^ M (Frankel et al., 2002a). Moreover, for seven different AML cell lines, IC_50_ values of less than 3 × 10^–11^ M were obtained. The ML-1 cell line was the most sensitive, with an IC_50_ of 5 × 10^–12^ M (Ramage et al., 2003).

To enhance the specific cytolethal efficacy of DTAT, a bispecific immunotoxin consisting of both the uPA amino-terminal fragment and IL-13 fused to DT390 was constructed (DTAT13). While *in vitro* and *in vivo* xenograft cytotoxicity assessments revealed very similar efficacy for the three immunotoxins, safety studies on intratumorally and intracranially treated xenograft mice indicated that DTAT13 was at least 160-fold less toxic than DTAT and at least eightfold less toxic than DTIL13 (a fusion of DT390 with IL-13) (Todhunter et al., 2004; Hall and Vallera, 2006).

On the basis of reports on the presence of EGFR on non-small cell lung cancer, a new DTAT derived immunotoxin, DTATEGF was constructed. *In vitro* anti-proliferative assessment of PC9-BrM3 (human brain metastatic lung cancer cell line) cells treated with DTATEGF revealed an IC_50_ of 1 × 10^–12^ M, compared to 1 × 10^–8^ M for DTEGF and 1 × 10^–9^ M for DTAT, representing a more than 1000- to 10000-fold increase in activity compared to each monospecific fusion toxin. Moreover, xenograft nude mice inoculated with the human PC9-BrM3 and treated with DTATEGF, showed significantly longer survival lengths compared to control mice. DTATEGF was administered intracranially via a micro-osmotic pump, and the mice received 1 μg of DTATEGF over 7 days (Huang et al., 2012).

Overall, the efficacy and anti-tumor effects of the three uPAR directed immunotoxins (DTAT, DTAT13, and DTATEGF) have been shown by different in *in vitro* and *in vivo* studies. Even with the mostly positive outcomes, there are no registered clinical trials or clinical evaluations for any uPAR-based immunotoxins.

### DTEGF13

DTEGF13 is a bispecific immunotoxin consisting of DT390 fused to EGF and IL-13, made to target a range of solid tumors including glioblastoma, prostate, and pancreatic cancers, which overexpress receptors for both EGF and IL-13. Used against different human cancer cell lines, including PC-3, DU-145 (prostate cancer cell lines) (Stish et al., 2007), U87MG, U118 (glioblastoma cell lines), calu-3 (human lung cancer cell line) (Stish et al., 2008; Oh et al., 2009), Panc-1, MiaPaCa-2 (pancreatic cancer cells), and H2981-T3 (lung adenocarcinoma cells) (Vallera et al., 2008), DTEGF has shown some of the lowest IC_50_s for immunotoxins studied to date, ranging from 4.2 × 10^–14^ M to 2 × 10^–11^ M. These values were around 905- to 2800-fold smaller, i.e., more toxic, than the corresponding monospecific DTEGF or DTIL13 immunotoxins.

In addition, separate *in vivo* studies in various established xenografts with flank PC-3, U87, or MiaPaCa-2 tumors revealed significant regression of the tumor masses and prolonged survival of the animals upon administration of four 2.5 μg intratumoral injections of DTEGF13. The fact that multiple injections of DTEGF13 were tolerated in the animals is important because human EGF and IL-13 are cross-reactive with mouse EGFR and IL-13R, thereby showing a higher specificity of DTEGF13 for tumor cells and lower safety concerns (Stish et al., 2008; Oh et al., 2009).

Further studies on DTEGF13 showed that a 200 μg bolus intraperitoneal (IP) dose of recombinant EGF13 a few minutes before administration of DTEGF13 in pancreatic cancer xenografted nude mice resulted in an over 15-fold increase in the MTD of the immunotoxin. With this “ToxBloc” strategy, the initial MTD was increased from 0.5 to 7.5 μg DTEGF13/injection (Oh et al., 2010). However, although this “ToxBloc” strategy showed to be efficient in widening the therapeutic window of the DTEGF13 immunotoxin, no further reports have been published regarding any preclinical or clinical study on this protein.

### DT-AntiCCR4

CC chemokine receptor 4 (CCR4) is expressed on the surface of some T cell-derived tumors including adult T-cell leukemia-lymphoma, adult peripheral T-cell lymphoma, T-cell acute lymphoblastic leukemia, and CTCL (Chang et al., 2012). In addition, this receptor is especially expressed by effector Treg cells, while it is absent on the surface of naïve Treg cells and, Th1 cells and is rarely expressed by CD8^+^ T cells, NK cells, CD14^+^ monocytes/macrophages, dendritic cells, and B cells (Sugiyama et al., 2013).

Various forms of DT-antiCCR4 fusions have been investigated, including monovalent DT390-scFv, bivalent DT390-biscFv, and DT390-Fold-back diabody with the aims of directly depleting both human CCR4^+^ tumor cells and CCR4^+^ Tregs as a combined cancer treatment. On the human CCR4^+^ acute lymphoblastic leukemia cell line, CCRF-CEM, the three variants (DT390-scFv, DT390-biscFv, DT390-Fold-back diabody) had IC_50_s of 2 × 10^–9^, 1 × 10^–10^, and 1 × 10^–11^ M, respectively. *In vivo*, DT390-biscFv and DT390-Fold-back diabody outperformed DT390-scFv. IL2 receptor γ^–/–^ NOD/SCID mice xenografted with CCRF-CEM cells were treated in two courses consisting of twice-daily administrations of 50 μg/kg of the three immunotoxins for 4 consecutive days. While significant survival was seen for DT390-biscFv and DT390-Fold-back diabody, DT390-scFv showed no improvement compared to the control (Wang et al., 2015). An *in vivo* study of two cynomolgus monkeys that received IV bolus of the fold-back diabody immunotoxin (25 μg/kg) twice a day for four consecutive days showed up to 89 and 96% depletion of their CCR4^+^ cells in the peripheral blood and lymph nodes, respectively. Other cell populations, including CD8^+^ T cells, other CD4^+^ T cells, B cells, and NK cells, were unaffected. However, due to the presence of CCR4 on the surface of other cell populations, e.g., CD4^+^CCR4^+^ and CD8^+^CCR4^+^ T-cells, further preclinical studies are necessary to evaluate their potential depletion (Wang et al., 2016).

### DT390-biTMTP1 and DT390-triTMTP1

Using bacterial display, a pentapeptide (NVVRQ) was isolated that specifically bound to highly metastatic cancer cells and yet had low affinity for poorly metastatic or non-metastatic tumor cells. Alone, this pentapeptide bound to prostate cancer PC-3M-1E8, breast cancer MDA-MB-435S, lung cancer PG-BE1, and gastric cancer MKN-45sci cell lines (Yang et al., 2008). As part of an immunotoxin, i.e., DT390-TMTP1, it showed little to no toxicity; however, DT immunotoxins with repeats of the pentapeptide, either as double or triple repeats (DT390-biTMTP1 or DT390-triTMTP1), were highly effective at killing PC-3M-1E8 and MKN-45 cells. In animal studies with established MKN-45 and PC-3M-1E8 tumor xenograft mouse models, injections of 10 μg DT390-biTMTP1 or DT390-triTMTP1 every 3 days for 21 days resulted in effective inhibition of subcutaneous tumor growth and prolonged mice survival (Ma et al., 2013). Despite the promising *in vitro* and *in vivo* results, no further preclinical studies on this immunotoxin have been reported.

### DT-AntiCD19

Using a previously reported sequence for anti-human CD19 scFV (Nicholson et al., 1997), three different DT390-antiCD19 immunotoxins have been investigated. The three immunotoxins consisted of DT390 fused to either a monovalent (scFV), bivalent (biscFV), or fold-back diabody fragment. In this case, cells were more sensitive to the biscFV than the fold-back diabody-based immunotoxin. On CD19^+^ JeKo (mantle cell lymphoma) cells, IC_50_s of 2 × 10^–10^, 1.7 × 10^–11^, and 2 × 10^–12^ M were obtained for scFV, fold-back diabody, and biscFV forms, respectively. The potency and safety of the immunotoxins were evaluated in a JeKo-cell xenografted immunodeficient NSG mouse model. Similar to the *in vitro* results, the bivalent form showed higher activity *in vivo*. Four courses of 100 μg/kg of each immunotoxin that was IP-injected twice daily for 4 consecutive days extended the survival rate of the mice by about 9 days (40-day survival post tumor induction) compared to negative controls (31-day survival post tumor induction). Given that no significant toxicity was reported in the animals, the dose could be increased further (Zheng et al., 2017). To date, no further studies on these immunotoxins have been reported.

### DT386-BR2

DT386-BR2 is a 47 KDa fusion protein containing the first 386 residues of DT fused via a rigid peptide linker, (AP)_4_, to the antimicrobial peptide, BR2 (Shafiee et al., 2016, 2017c). BR2 is derived from bufoin IIb (RAGLQFPVG[RLLR]_3_), a potent antimicrobial peptide with cell-penetrating and anti-cancer properties (Lee et al., 2008; Cho et al., 2009). BR2 has only two terminal RLLR repeats and is selective in its ability to penetrate and kill cancer cells without affecting normal cells (Lim et al., 2013; Shafiee et al., 2017b). *In vitro* studies showed that DT386-BR2 was cytotoxic against HeLa (cervical carcinoma) and MCF-7 (breast cancer) cells, with little toxicity to HEK 293 and HUVEC cells (Shafiee et al., 2016). Compared to other immunotoxins, however, the reported IC_50_s between 10^–8^ and 4 × 10^–8^ M against HeLa and MCF-7 are relatively high. Still, intraperitoneal injection into healthy albino mice in a preclinical safety study revealed no significant non-specific toxicity. The LD_50_ was greater than 10 mg/kg (Shafiee et al., 2017a), and permission for further preclinical study on xenograft human tumors has been granted.

### DT387-SCF

Stem cell factor (SCF) is a hematopoietic growth factor promoting the survival, proliferation, and differentiation of hematopoietic stem cells and progenitor cells by itself or in synergy with other cytokines (Brandt et al., 1992). Overexpression of its receptor, c-kit, has been reported in several cancers such as liver (Chung et al., 2005), ovarian (Raspollini et al., 2004), stomach (Hirota et al., 1998), pancreatic (Yasuda et al., 2006), and small cell lung carcinoma (Naeem et al., 2002). DT387-SCF targets c-kit-overexpressing malignancies. It has been shown to be cytotoxic against PANC-1 (pancreatic carcinoma), MOLT4 (acute lymphoblastic leukemia), HeLa, and K562 (chronic myelogenous leukemia) cells, with IC_50_s ranging from 4 × 10^–7^ to 8.8 × 10^–7^ M. HuT78 (Human T-cell lymphoma) cells were unaffected by DT-SCF. Although DT387-SCF showed moderate cytotoxicity against certain cells, the high IC_50_ values may be one of the reasons that there have been no further reports on this fusion protein (Potala and Verma, 2010).

### DT-Gastrin-Releasing Peptide

Based on various studies showing that the gastrin-releasing peptide (GRP, an autocrine growth factor) receptor is up-regulated on the surface of different malignant cell types including cell lines from small cell lung, breast, prostate, and colon cancers (Carney et al., 1987; Schutte and Seeber, 1993), an immunotoxin with GRP has been produced (DT389-GRP). The lowest IC_50_ of this fusion protein on small cell lung cancer cells tested was for NCI-H345, a cell line with the highest levels of GRP receptor expression. The IC_50_ was recorded as 1.1 × 10^–9^ M (Vanderspek et al., 1997). Despite a good correlation between levels of GRP and toxicity, no further studies have been published.

### Immunogenicity of DT-Based Immunotoxins

Because the general population is usually vaccinated against diphtheria, most patients have pre-existing antibodies against the toxin. These antibodies can neutralize the immunotoxin and result in lower treatment efficiency. Even if a patient does not have a pre-existing immunity, treatment schedules that require multiple courses can suffer from the production of antibodies against the toxin moiety during the treatment (Frankel et al., 2003, 2009; Bachanova et al., 2015). Attempts have been made to deimmunize toxin moieties. The highly hydrophilic amino acids glutamine, arginine, lysine, aspartic acid, and glutamic acid have been shown to be highly immunogenic in different studies (Onda et al., 2006). Point mutations of these highly hydrophilic amino acids present on the molecular surface of truncated diphtheria (DT390) have led to lower levels of antibodies when the mutant form of the toxin has been used as the toxin moiety. In a series of steps, it has been shown that up to seven mutations (K125S, R173A, Q245S, K385G, E292S, Q184S, and K227S) could be made to the toxin moiety (dDT) of dDTEGF13 without losing more than a log of activity (Schmohl et al., 2015). In an animal study consisting of 12 injections (on days 0, 1, 14, 21, 28, 35, 42, 49, 56, 63, 70, 77) and evaluated on day 84, dDTEGF13 elicited only a minimal antibody response, while DTEGF13 had an average anti-DT390 response of greater than 1500 μg/mL (Schmohl et al., 2015).

Similarly, DT390 was replaced by dDT390 in DT2219 to form dDT2219. The newly engineered immunotoxin was tested for activity, efficacy, and specificity using functional assays, proliferation assays, and flow cytometry. In addition, the immunogenicity of the molecule was evaluated in BALB/c mice. dDT2219 induced significantly lower levels of neutralizing antibodies compared to DT2219 without compromising its specific cytholethal activity on two different CD22^+^CD19^+^ Burkitt-lymphoma cell lines (Schmohl et al., 2018).

## Expression of Dt Under the Control of Specific Promoters

**FIGURE 4:**
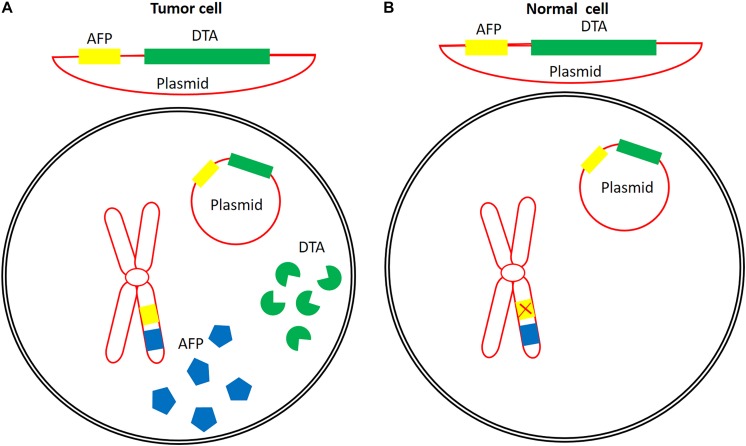
An example of the concept of the application of a tumor-specific promoter for tumor-specific expression of DTA. Alpha feto-protein promoter is active in some cancers **(A)**, but is inactive in normal cells **(B)**. Construction of a plasmid containing the DTA coding sequence under the control of this promoter might lead to the expression of DTA in tumor cells with active AFP promoter without any expression in or cytotoxic effects on normal cells. Eukaryotic chromosome (red), AFP (blue), AFP promoter (yellow), and DTA (green).

To bypass issues related to the production, purification, formulation, administration, and immunogenicity of DT-based immunotoxins, the genetic material that codes for the catalytic domain of DT (DTA) can be delivered instead. Expression of the gene *in vivo* can then be controlled using tumor-specific promoter sequences. Figure 4 schematically illustrates the mechanism of tumor-specific expression of DTA.

### H19 Regulatory Sequence

H19 is a non-protein-coding onco-fetal gene that acts as a riboregulator upon transcription. It is highly expressed in different tumor types including hepatocellular (Ariel et al., 1998), bladder (Ariel et al., 2000), and chorio- (Lustig-Yariv et al., 1997) carcinoma, in addition to colorectal (Cui et al., 2002) and ovarian (Tanos et al., 1999) cancers, but its expression in normal tissues is very low or undetectable (Kaplan et al., 2003). Using the 814 bp flanking the 5′-region of the H19 gene, expression of DTA was assessed based on the inhibition of luciferase activity when various quantities of pDTA-PBH19 were co-transfected with a set amount of pLuc4SV40 reporter plasmid (7 μg). A potent decrease in luciferase activity was observed in T24P bladder carcinoma cells. Using 0.05 to 1 μg/well of pDTA-PBH19 resulted in 30 to 90% inhibition, respectively. In IMR-90 cells, where the H19 regulatory element is inactive, no significant reduction in luciferase activity was observed. Substantial reductions in luciferase activity (about 85%) were also seen in Hep3B (human hepatocellular carcinoma) and MBT-2-t50 (mouse bladder carcinoma) cells, albeit with 0.5 μg/well of pDTA-PBH19 (Ohana et al., 2002). The anti-tumor potential of pDTA-PBH19 was assessed in a murine bladder carcinoma model established by subcutaneous injection of MBT-2-t50 cells. Intra-tumor administration of three 50 μg doses of pDTA-PBH19 as a calcium phosphate precipitate resulted in a 40% reduction in tumor size compared to the control groups, which received either the pLuc-PBH19 reporter plasmid or no treatment (Ohana et al., 2002). In a subsequent pilot clinical study, the safety and efficacy of the pDTA-PBH19 plasmid were evaluated in two human patients having recurrent superficial bladder carcinoma refractory to common treatments and whose cancer cells showed high levels of H19 RNA. A dose of 2 mg of the plasmid was delivered intravesically once a week for a total of 9 weeks. A reduction in tumor size of 75% without any significant adverse effects or traces of pDTA-PBH19 in their blood was seen for both patients (Ohana et al., 2004). pDTA-PBH19, now under the name BC-819, was subsequently evaluated in a phase I/II dose-escalation clinical trial on 18 patients with superficial bladder cancer refractory to intravesical therapy with the Bacillus Calmette–Guerin (BCG) vaccine. The patients received weekly intravesical doses of 2, 4, 6, 12, or 20 mg over 7 weeks. The doses were escalated if none of the first three patients in the preceding dose cohort experienced dose-limiting toxicity after the first three weekly intravesical treatments. Of the 18 patients, four went into CR and eight showed either complete ablation of marker tumors or at least a 50% reduction in marker lesions by week 12. No dose-limiting toxicity or death was observed during the study, confirming the safety of BC-819 (Sidi et al., 2008). The safety and efficacy results were also further confirmed by a phase IIb prospective, open-label, multicenter trial on patients with recurrent, multiple non-muscle-invasive bladder cancer with prior intravesical therapy failure. In that study, patients received intravesical instillations of six 20 mg plasmid DNA over 6 weeks. Following this period, patients were given 3-weekly maintenance treatment every 3 months for up to 1 year. Of the 39 evaluable patients, 64% were recurrence-free at 3 months. At 1 and 2 years, the recurrence-free rate was 45 and 40%, respectively. In addition, only one drug-related severe adverse effect was observed; one patient developed hematuria (Gofrit et al., 2014).

The anti-tumor potential of BC-819 was also evaluated in ovarian cancer cell lines. Co-transfection of 2 μg pLucSV40/well and 0.0125 μg pDTA-H19/well in OVCAR-3, TOV-112D, and ES-2 resulted in 30, 75, and 55% reductions in luciferase activity, respectively, compared to the controls. *In vivo* administration of four 25 μg injections of pDTA-H19 every other day to ES-2 cell line-xenografted nude mice inhibited tumor growth by 40% (Mizrahi et al., 2009). In a human case, an 80 mg intraperitoneal instillation of this construct to a woman with stage IIIc epithelial ovarian cancer showed no adverse effects. Gradually increasing the dose to 140 mg up to the 7th week led to complete resolution of the ascites and malignant cells with minimal adverse effects (Mizrahi et al., 2010a).

The synergistic activity of DTA and TNFα on ovarian tumor cell lines and nude mice ovarian cancer models has also investigated. Expression of the two proteins under the control of the H19 promoter (pH19-TNF-IRES-DTA) occurred via an internal ribosomal entry site (IRES) element located at the 5′ end of the DTA fragment. Co-transfection of ES-2, TOV-122D, and SK-OV3 cell lines with 2 μg/well of a pLucSV40 and 0.025 μg/well of the pH19-TNF-IRES-DTA plasmid reduced the luciferase activity by 96%. The IC_50_ values for pH19-TNF-IRES-DTA on ES-2 and TOV-122D cells were lower than those for pH19-DTA on the same cell lines (0.0025 and 0.003 μg pH19-TNF—IRES-DTA/well for ES-2 and TOV-122D versus 0.004 μg pH19-DTA/well for both cell lines). Even the DTA- and TNF-resistant SK-OV3 cells were efficiently killed (IC_50_ value of 0.004 μg plasmid/well) using the pH19-TNF-IRES-DTA plasmid. In addition, 25 μg intra-tumoral injections of pH19-DTA or pH19-TNF-IRES-DTA into ectopically grown human ovarian tumors in athymic female nude mice inhibited tumor growth by 40% (Mizrahi et al., 2010b).

Further *in vitro* and *in vivo* preclinical studies also revealed potential for application of the pDTA-PH19 plasmid in the treatment of colorectal adenocarcinoma, lung cancer, and pancreatic cancer, alone or in combination with the chemotherapeutic agent gemcitabine (Hasenpusch et al., 2011; Sorin et al., 2011, 2012). Furthermore, an open-label, dose-escalation phase I/II clinical trial on nine patients with unresectable pancreatic adenocarcinoma showed promising results. The MTD for patients receiving a 4–8 mg intratumoral injection of the plasmid twice weekly for 2 weeks was greater than 8 mg/injection. Moreover, in addition to decreased tumor size in all of the patients, four patients who received the highest amount of the plasmid survived for more than 6 months, and 2 others survived for more than 12 months (Hanna et al., 2012). Recently, a phase I/IIa study on 14 patients with recurrent, platinum-resistant advanced-stage ovarian cancer or primary peritoneal carcinoma showed mixed results. The first cohort received a dose of 60 mg of BC-819 intraperitoneally, while subsequent cohorts received escalating doses of 120 and 240 mg. Results revealed only five drug-related adverse effects in four patients in the 60 and 120 mg cohorts, and no adverse effect was reported for the 240 mg cohort. Unfortunately, the overall survival seen in that study was between 6.3 and 15 months, which is similar to the median overall survival rate in heavily pretreated patients with recurrent platinum-resistant ovarian cancers. It has been suggested that a larger cohort study, with higher doses and longer periods of treatment in combination with other systemically administered drugs, is needed (Lavie et al., 2017).

### Insulin-Like Growth Factor 2, Promoters 3 and 4

The human insulin-like growth factor 2 (IGF2) gene is transcribed under the control of four different promoters, P1–P4, which results in four different transcripts (Holthuizen et al., 1990). The P3 and P4 promoters are only active during embryogenesis and in some tumor tissues (Engstrom et al., 1998). Ayesh et al. (2003) verified the activity of these promoters in T24P (human bladder carcinoma), HepG2, and Skhep1 (hepatocellular carcinoma) cell lines using a reporter plasmid harboring the luciferase gene downstream of the P3 or P4 promoters. Their results confirmed the activity of both promoters in HepG2 and T24P cell lines; however, in Skhep1 cells, the promoters had limited functionality. When the same cells were co-transfected with plasmids bearing DTA under the control of either P3 (pP3-DTA) or P4 (pP4-DTA) promoters and pLucSV40 (a plasmid that allows constitutive expression of luciferase), a dose-dependent response was seen in all three cell types. A 50% reduction in luciferase activity was obtained following transfection of 0.01 and 0.015 μg/well pP3-DTA in HepG2 or T24P cells, respectively. In the same cells, transfecting with pP4-DTA only required 0.005 μg/well of the plasmid to achieve the same level of luciferase inhibition. However, for both P3-DT-A and P4-DT-A, higher concentrations were needed to achieve a 50% reduction in Skhep1.

Human P3 and P4 promoters are also active in the murine MBT-2-t50 bladder carcinoma cell line. To further test pP3-DTA, an animal model of the carcinoma was established by subcutaneous injection of the cells and treated by three 50 μg intratumoral injections of pP3-DTA over 5 days. Tumor growth was significantly inhibited (70%) compared to non-treated mice or mice injected with a LucP3 plasmid (*P* < 0.0002). In addition, a group of healthy animals receiving the same doses of pP3-DTA plasmid did not show any signs of toxicity after 24 days and continued to gain weight, similar to the healthy animals injected with LucP3. Furthermore, the histopathological examination of the two groups did not reveal any evidence for toxicity in any of their internal organs (liver, kidney, heart, spleen, pancreas, and adrenals) (Ayesh et al., 2003). To further improve the efficiency of these plasmids, Amit et al. (2011) developed a double-promoter plasmid by joining the two DTA-expressing cassettes. *In vitro* evaluation of the dual-cassette plasmid (pP4-DTA-P3-DTA) in various cancer cell lines showed that up to 70% inhibition of luciferase activity could be achieved with as little as 0.005 μg of plasmid preparation per well (Amit et al., 2013). *In vivo*, 3 doses of 25 mg intratumoral injections of the plasmids into a murine model of human bladder carcinoma over 5 days reduced tumor size by 70, 45, and 50% using pP4-DTA-P3-DTA, pP3-DTA, and p4-DTA, respectively (Amit et al., 2011). Despite the promising results *in vitro* and *in vivo*, there is no indication that these plasmids have progressed to preclinical or clinical studies.

### Rad51, Rad51C, and XRCC2 Regulatory Elements

The Rad51 protein is involved in homologous recombination and has an essential role in repairing DNA double-strand breaks. The expression of Rad51 in normal cells is tightly controlled, but in a variety of tumors, it can be overexpressed (Raderschall et al., 2002). Hine et al. (2008) constructed a plasmid (pRAD51-DTA) expressing DTA under the control of the 6,532-bp Rad51 regulatory region and tested it on a panel of 13 normal and malignant cells. Cells were co-transfected with 0.01–0.1 μg of pRAD51-DTA together with a plasmid-bearing luciferase under the control of a constitutive promoter. All the tumor cell lines except MDA-MB-468 experienced a 10–100,000-fold decrease in protein synthesis. In normal cells, decreases in protein synthesis were less than 10-fold. In addition, transfection with pRAD51-DTA resulted in a 20–80% reduction in viability in all of the cancer cell lines (HeLa, MCF-7, T47-D, HCC1954, GP2-293, HT1080, and MDA-MB-468) and had little to no effect on normal cell lines (HMEC-1, HMEC2, HMEC4, IMR90, WI-38, and HCA2).

*In vivo* effects of this construct were investigated in nude mice xenografted with human cervical cancer cells. The xenograft tumors were made by IP or SC injection of HeLa cells to the nude mice. The effect of the Rad51 controlled expression of DTA was evaluated following IP injections of Rad51-DTA plasmid in the case of IP tumors (receiving 6 doses of 100 μg plasmid over 15 days) or intratumoral injections to SC tumors (receiving 6 doses of 20 μg plasmid over 28 days). In the case of SC tumors, 50 percent of the mice were cancer-free after 6 doses of the plasmid injection. In addition, for intra-peritoneal tumors, IP administration of the plasmid resulted in a 90% increase of mean survival time compared to the controls (Hine et al., 2012).

Despite the promising cancer cell-selective activity of the pRAD51-DTA, its application was hindered due to the large size of the Rad51 regulatory element (6532 bp). Therefore, attempts were made to examine the tumor specificity of Rad51 paralogs having shorter regulatory nucleotide sequences. Cao et al. (2014) evaluated the expression level and specificity of four Rad51 paralogs, including Rad51B, Rad51C, Rad51D, and Rad52, with a panel of 14 different malignant and normal cell lines. Among these paralogs, Rad51C, with a ∼2000 bp length regulatory sequence, showed ninefold higher protein expression in cancer cells. In addition, transfection of normal and malignant cells by a plasmid driving DTA expression by the Rad51C promoter (pRAD51C-DTA) resulted in 7- to 10- fold greater inhibition of luciferase production and activity in cancer cells compared to normal cells (Cao et al., 2014). The use of XRCC2, another Rad51 paralog with a shorter regulatory sequence (∼2100 bp), resulted in an up to 90% reduction in luciferase activity in cancer cells, while only causing an up to 19% reduction in normal cells. The cytolethal effects after transfection of pXRCC2-DTA and pRAD51-DTA into HeLa cells were very similar (76.7 vs. 82.1%, respectively). Moreover, *in vivo* evaluation in nude mice, subcutaneously xenografted with HeLa cells, which consisted of 4 intratumoral injections of lentiviral vector containing the XRC22-DTA cassette in 11 days, resulted in a significant reduction in tumor size (Chen Y. et al., 2018).

### Prostate-Specific Antigen Promoter

The promoter and upstream sequences (PSAR-PCPSA promoter) of human prostate-specific antigen (PSA) have been used to drive DTA expression in PSA-positive prostate cancer cells using the pPSAR-PCPSA-DTA plasmid. However, although liposome-based transfection of LNCaP (PSA positive prostate), DU145 (non-PSA-producing prostate), and R11 (renal) cell lines reduced the viability of LNCaP cells without significant effect on R11 and DU145 cells, the transfection efficiency was low (Pang, 2000). To overcome the low transfection efficiency, a lentiviral vector carrying the PSAR-PCPSA-DTA expression cassette was prepared and used to infect LNCaP and different PSA-negative control cell lines. Infection of the cells with MOIs of 0.5 or higher resulted in a 50–95% reduction in LNCaP cell viability but did not affect the control cell lines (Yu et al., 2001). In addition, intratumoral injection of the prepared lentiviral vectors in nude mice subcutaneously xenografted with LNCaP cells resulted in significant tumor regression 4–7 days post-injection, without any pathogenic effects even up to 90 days (Zheng et al., 2003).

Peng et al. (2005) also developed a viral vector-mediated approach using the PSA promoter. Using an adenoviral vector, they were able to show significant inhibition of protein synthesis by measuring luciferase enzyme activity in the LNCaP cell line. Furthermore, subcutaneous LNCaP tumors in nude mice were reduced in size by 50% upon intratumor injection of the adenoviral particles. This was also observed by intratumoral injection of the adenoviral particles into tumors of TRAMP mice, transgenic mouse models of prostate tumors. Taken together, the promoter sequence of PSA showed to be effective in tumor-specific expression of DTA fragments for the treatment of cancer.

### Survivin Promoter

Survivin is type of inhibitor of the apoptosis protein family (Ambrosini et al., 1997), which is expressed in multiple types of cancers including ovarian (Chen et al., 2013), gastric (Meng et al., 2013), non-small- (Xu et al., 2012) and small-cell (Chen et al., 2014) lung, and breast cancer (Tanaka et al., 2000) but is undetectable in normal tissues (Chen et al., 2004). Evaluation of two different survivin promoter fragments, Surp269 and Surp1430 (269 or 1430 bp, respectively), to drive luciferase expression showed much higher activity for the longer fragment, Surp1430, in three different cancer cell lines (HeLa, Eca109, and ZR-7530). A lentiviral vector harboring a Surp1430-DTA expression cassette was used to transduce various normal and malignant cell lines. With 0.1 and 0.3 μg of the recombinant virus, a 42 to 92% reduction in cell viability was observed for the malignant cells, while the viability of normal cells was not affected. Furthermore, when nude mice xenografted with a ZR7530 breast cancer cell line were subjected to the Surp1430-DTA lentiviral vector 30, 40, and 50 days after implantation of the cells, significant regression of tumor masses was observed. However, the tumors were not completely eradicated, and a very small mass of tumors survived (Lin et al., 2015). This incomplete tumor mass regression, in addition to findings on survivin protein overexpression in megakaryocyte and erythrocytes and its up-regulation by β-catenin, has impeded further application of this promoter for tumor-targeted gene expression (Mccrann et al., 2008).

### Alpha-Fetoprotein Promoter

Alpha-fetoprotein (AFP) is expressed during fetal development; however, after birth, its expression is negligible except in the case of certain malignancies including hepatocellular carcinomas and teratomas, where AFP is expressed at high levels (Chen et al., 2007). In one study, a plasmid containing DTA linked to the human AFP promoter and enhancer (pAF-DTA) was investigated for its selective cytolethal effects on AFP-producing cells HuH-7 and HepG2. Cells were co-transfected with both pAF-DTA and a plasmid bearing the chloramphenicol acetyltransferase (CAT) gene (pAF-CAT). CAT activity decreased with increasing amounts of pAF-DTA from 0.5 to 3 μg/10^6^ cells. Transfection of pAF-DTA in HuH-7 and HepG2 cells inhibited their growth, whereas following transfection of the AFP-negative cell line, MKN45, no growth inhibition was observed (Murayama et al., 1999). Furthermore, intra-tumor injection of the pAF-DTA plasmid in a hepatocellular carcinoma nude mouse model xenografted with HuH-7 tumors resulted in significant tumor weight reduction (0.62 ± 0.21 g) compared to PBS treated controls (2.07 ± 0.67 g) (Kunitomi et al., 2000). These results are promising for the treatment of cancerous cells expressing AFP without affecting other types of cells.

### hTERT Promoter

Telomerase activity can be detected in about 85% of different malignant tumors but is absent in most normal cells (Ito et al., 1998). Therefore, human telomerase transcriptional regulatory sequences, hTER (responsible for the transcription of the RNA subunit of the human telomerase) and hTERT (responsible for transcription of the human telomerase reverse transcriptase subunit) have been targeted to drive the expression of DTA. These two regulatory sequences were tested by co-transfecting plasmids (phTER-DTA or phTERT-DTA) with a luciferase reporter plasmid in various cell lines with high-level telomerase activity (RT112 (bladder carcinoma) and HepG2 (hepatocellular carcinoma) cells), low-level telomerase activity (U2OS and Saos-2 osteosarcoma cells), or no telomerase activity (IMR-90 cells, a primary fibroblast cell line). A reduction in luciferase activity was observed in all of the malignant and normal cell lines, showing non-cancer cell specificity of hTER. However, highly tumor-specific reduction rates (82 to 55%) were observed for the phTERT-DTA, even in the low telomerase activity osteosarcoma cell lines, compared to the IMR-90 control cells (which had an up to 20% reduction). These results confirmed hTERT as a promising promoter for gene therapy applications (Abdul-Ghani et al., 2000).

### Human Chorionic Gonadotropin α and β Promoters

Human chorionic gonadotropin (hCG) hormone is a glycoprotein composed of two non-covalently bound α and β subunits. Normally, this hormone is a sign of pregnancy and can be detected in the blood and urine of pregnant women; however, in cases of breast and ovarian cancer, free hCG α or β subunits can also be detected. In certain cancers, there is activation of the regulatory sequences controlling transcription of the hCG subunits (Cole et al., 1988). Therefore, to test if DTA could be preferentially expressed in cancer cells, plasmids were constructed that had DTA downstream of α or β hCG promoters (creating pTHA-47 or pTHA-49 plasmids, respectively). Co-transfecting these plasmids (individually) with a second plasmid harboring luciferase downstream of a constitutive promoter, the expression of DTA could be assessed through the inhibition of luciferase activity. Transfection into EBLY II, OVCAR-V, and OVCAR III cells resulted in up to 95% inhibition of luciferase compared to the control. Of the two promoters, the α subunit promoter yielded greater inhibition than the β subunit promoter. Unfortunately, luciferase inhibition, albeit small, was also seen when the plasmids were transfected into a normal ovarian cell line, thereby restricting further applications of these promoters (Lidor et al., 1997).

### β-Catenin-Dependent Promoter

β-catenin is a component of cadherin-based adherens junctions. Deregulation in its structure and signaling properties often results in deregulated growth connected to cancer and metastases (De La Coste et al., 1998; Valenta et al., 2012). Lipinski et al. (2001) developed artificial β-catenin-dependent promoters that are highly active in cancer cells *in vitro* and *in vivo*. An adenovirus vector carrying DTA under the control of CTP4, an optimized β-catenin-dependent artificial promoter, was used to show a dose-dependent (adenoviral MOI) decreased cell survival in the β-catenin-deregulated tumor cell lines SW480 and HepG2 compared to control cells with regulated β-catenin activity, such as HeLa, HNX14C, and HUVEC (Lipinski et al., 2004).

### Delivery of the DTA-Expressing Cassettes

In order to deliver DTA-expressing cassettes to cancer cells, different strategies, including the application of naked plasmids or viral vectors, have been considered. Usually, to improve cellular uptake of the intratumorally injected naked plasmids, they are prepared in a mixture of *in vivo* safe transfection reagents such as calcium phosphate (Ohana et al., 2002) or *in vivo*-jetPEI^TM^ (Hine et al., 2012). In some cases, delivery is enhanced using adeno- (Li Y. et al., 2002) or lenti- (Lin et al., 2015) viral vectors. One problem with preparation of DTA-encoding viral vectors is the leaky expression of DTA in packaging cell lines, which disturbs the host cell protein synthesis machinery and lowers viral particle titers or even prevents viral particle formation. To alleviate this problem, DT-resistant packaging cell lines such as HEK293T[mEF-2(G717R) (Mccrann et al., 2008) or 293DTRP#2 (Li Y. et al., 2002) were developed. Another strategy proposed to lessen the problem of leaky expression, especially in normal tissues, is using a two-plasmid (binary) system, in which one plasmid carries a silenced DTA gene separated from its promoter by a transcription terminator, and the second plasmid carries the encoding sequence for site-specific Cre recombinase under a cancer-specific promoter. Once inside the cancer cells, the recombinase removes the transcription terminator sequence flanked by attR and attL recombination sites and activates the expression of DTA. This could result in less DTA expression in normal cells and lower non-specific toxicity (Elias et al., 2018).

## Conclusion

Diphtheria toxin is lethal and, without a targeted approach, significant toxicity can be experienced by the patient. In fact, certain clinical trials had to be cut short or failed once completed because of these effects. It is for this reason that strategies that can harness the power of the toxin while minimizing off-target effects are consistently being sought. It is clear that there is great potential for the catalytic domain of DT. However, this potential is not new, and this review highlights work that dates back as early as the 1980s. Two broad approaches have been used for taming this toxin into serving as a therapeutic agent: direct toxin delivery or delivery of the gene for the production of the toxin *in vivo.* Amongst all of the examples described above, the only approved agents that reached the market are Ontak^TM^ and Tagraxofusp^TM^. Some immunotoxins and gene-based constructs are still in pre-clinical and clinical trial stages, without any final decision on their overall clinical applicability. The development of more specific targeting strategies for the protein could result in fewer adverse and non-expected effects on normal cells and tissues. Moreover, the development of methods for enhanced delivery of the vectors for gene-based therapy with DTA should result in higher efficiency in eradicating tumor cells. However, in addition to the targeting and delivery issues with the DTA-based therapies, which are also common to other bacterial or plant toxins, is a preexisting immunity of the general population due to existing immunization schemes. This preexisting immunity could result in lower therapeutic responses or even clearance of the DT-fusion proteins by the patient immune system. This may give an advantage to gene-based DT therapeutics going forward.

## Author Contributions

FS, MA, and AJ-N took part in drafting, revising, and preparation of the manuscript. The manuscript was finalized and prepared for submission by AJ-N. Further revisions according to the reviewer comments were made by MA and AJ-N and finalized by AJ-N.

## Conflict of Interest

The authors declare that the research was conducted in the absence of any commercial or financial relationships that could be construed as a potential conflict of interest.

## Abbreviations

ADPR: adensosine diphosphate ribosyl
AML: acute myeloid leukemia
BPDCN: blastic plasmacytoid dendritic cell neoplasm
CCP: cell-penetrating peptide
CR: complete remissions
CTCL: cutaneous T-cell lymphoma
DT: diphtheria toxin
EGF: epidermal growth factor
GM-CSF: Granulocyte macrophage-colony stimulating factor
HD: Hodgkin’s disease
MTD: maximum tolerated dose
NAD: nicotinamine dinucleotide
NHL: non-Hodgkin’s lymphoma
PR: partial remissions
PTCL: peripheral T-cell lymphoma.
